# *cis*-9,10-Bis(bromo­meth­yl)-1,4,5,8-tetra­oxadeca­lin

**DOI:** 10.1107/S1600536808015377

**Published:** 2008-05-30

**Authors:** Courtney L. Yambo, Patrick S. Williams, David J. Au, Daniel S. Jones, Markus Etzkorn

**Affiliations:** a Department of Chemistry, The University of North Carolina at Charlotte, 9201 University City Blvd., Charlotte, NC 28223, USA

## Abstract

The title compound, C_8_H_12_Br_2_O_4_, is a bicyclic ketal in which the two six-membered rings are *cis* to one another and assume a double-chair conformation. A crystallographic twofold axis bisects the molecule.

## Related literature

A determination of this structure has been previously attempted (Fuchs *et al.*, 1972 ▶), but the authors did not publish or deposit any atomic coordinates. The same paper gives detailed information for a related structure which has H atoms in place of the bromo­methyl groups of the title compound. A search of the Cambridge Structural Database [Version 5.29; (Allen, 2002 ▶); *CONQUEST* (Bruno *et al.*, 2002 ▶)] did not yield any other closely related structures. See also: Fuchs (1970 ▶).
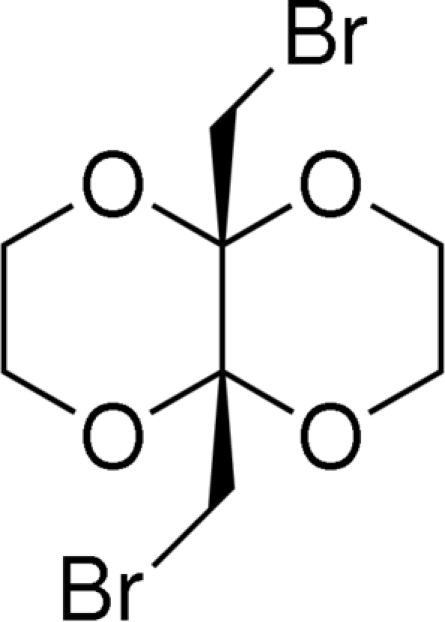


## Experimental

### 

#### Crystal data

C_8_H_12_Br_2_O_4_
*M* *_r_* = 332
Orthorhombic, 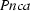

*a* = 8.5314 (14) Å
*b* = 9.5295 (7) Å
*c* = 13.1348 (13) Å
*V* = 1067.9 (2) Å^3^
*Z* = 4
Cu *K*α radiation
μ = 9.57 mm^−1^
*T* = 295 (2) K
0.5 × 0.3 × 0.15 mm

#### Data collection

Enra–Nonius CAD4 diffractometer
Absorption correction: multi-scan (Blessing, 1995 ▶) *T*_min_ = 0.039, *T*_max_ = 0.486 (expected range = 0.019–0.238)
4584 measured reflections
968 independent reflections
915 reflections with *I* > 2σ(*I*)
*R*_int_ = 0.063
3 standard reflections every 67 reflections intensity decay: 3%

#### Refinement

*R*[*F* ^2^ > 2σ(*F* ^2^)] = 0.036
*wR*(*F* ^2^) = 0.101
*S* = 1.10
968 reflections
65 parameters
H-atom parameters constrained
Δρ_max_ = 0.45 e Å^−3^
Δρ_min_ = −0.43 e Å^−3^

### 

Data collection: *CAD-4 EXPRESS* (Enraf–Nonius, 1994 ▶); cell refinement: *CAD-4 EXPRESS*; data reduction: *XCAD4* (Harms & Wocadlo, 1995 ▶); program(s) used to solve structure: *DIRDIF* (Beurskens *et al.*, 1999 ▶); program(s) used to refine structure: *SHELXL97* (Sheldrick, 2008 ▶); molecular graphics: *ORTEP-3 for Windows* (Farrugia, 1997 ▶); software used to prepare material for publication: *WinGX* (Farrugia, 1999 ▶).

## Supplementary Material

Crystal structure: contains datablocks global, I. DOI: 10.1107/S1600536808015377/fj2094sup1.cif

Structure factors: contains datablocks I. DOI: 10.1107/S1600536808015377/fj2094Isup2.hkl

Additional supplementary materials:  crystallographic information; 3D view; checkCIF report

## Figures and Tables

**Fig. 1.**
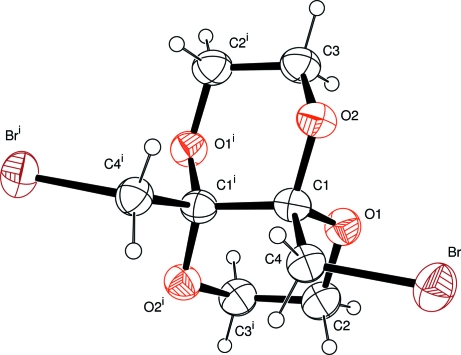
View of the title compound, 3, with 50% probability displacement ellipsoids. [Symmetry code: (') -x + 1/2, -y + 1, z.]

**Fig. 2.**
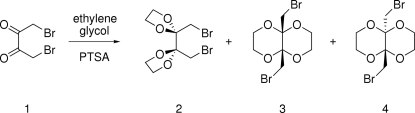
Schematic representations of compounds 1–4.

## supplementary crystallographic information

### Comment

The title compound is a bicyclic ketal in which the two six-membered rings are *cis* to one another and assume a doublechair conformation. A crystallographic twofold axis relates one of the fused rings to the other.

A determination of this structure has been previously attempted (Fuchs *et al.*, 1972), but the authors did not publish or deposit any atomic coordinates. They halted their study after three cycles of isotropic refinement because "···the accuracy of the atomic parameters was relatively low." They did present the Fourier map resulting from the isotropic refinement, and it shows an overall structure in agreement with that presented here. The space group and unit-cell parameters which they obtained are also in agreement with those of the present study.

The authors of this prior study attributed the "relatively low" accuracy of their atomic parameters to the domination of the scattering by the Br atoms. Our present study suggests that the importance of absorption corrections probably played a part as well.

The same paper (Fuchs *et al.*, 1972) gives detailed structural information for a related compound which has hydrogen atoms in place of the bromomethyl groups of the title compound. That structure is similar to the one reported here, in that the two six-membered rings are *cis* to one another and assume a doublechair conformation.

### Experimental

The title compound 3 has been described as the major reaction product in the acid-catalyzed reaction of diketone 1 with ethylene glycol (Fuchs, 1970). Since the dispiro derivative 2 was needed for one of our projects, literature procedures (Fuchs *et al.*, 1972) were followed to yield a product mixture from which only the bicyclic title compound 3 could be isolated, as colorless crystals. The NMR data obtained for 3 deviated from the published data (Fuchs, 1970), and the X-ray structure was therefore determined to ultimately confirm the formation of 3.

^1^H NMR (CDCl_3_, 500 MHz): δ = 4.14 (m_c_, 4H), 3.75 (m_c_, 4H), 3.69 (s, 4H) p.p.m..

^13^C NMR (CDCl_3_, 125.7 MHz): δ = 92.3, 61.1, 31.6 p.p.m..

### Refinement

All H atoms were constrained using a riding model. C — H bond lengths were fixed at 0.97 Å, with *U*_iso_(H) = 1.2 U_eq._ (C).

### Crystal data

**Table:**

C_8_H_12_Br_2_O_4_	*F*_000_ = 648
*M**_r_* = 332	*D*_x_ = 2.065 Mg m^−^^3^
Orthorhombic, *P**n**c**a*	Cu *K*α radiation λ = 1.54184 Å
Hall symbol: -P 2a 2n	Cell parameters from 23 reflections
*a* = 8.5314 (14) Å	θ = 9.7–23.5º
*b* = 9.5295 (7) Å	µ = 9.57 mm^−^^1^
*c* = 13.1348 (13) Å	*T* = 295 (2) K
*V* = 1067.9 (2) Å^3^	Irregular, colorless
*Z* = 4	0.5 × 0.3 × 0.15 mm

### Data collection

**Table:**

Enra–Nonius CAD4 diffractometer	*R*_int_ = 0.063
Radiation source: normal-focus sealed tube	θ_max_ = 67.6º
Monochromator: graphite	θ_min_ = 5.7º
Non–profiled ω/2θ scans	*h* = −10→10
Absorption correction: multi-scan(Blessing, 1995)	*k* = −11→0
*T*_min_ = 0.039, *T*_max_ = 0.486	*l* = −15→15
4584 measured reflections	3 standard reflections
968 independent reflections	every 67 reflections
915 reflections with *I* > 2σ(*I*)	intensity decay: 3%

### Refinement

**Table:**

Refinement on *F*^2^	H-atom parameters constrained
Least-squares matrix: full	*w* = 1/[σ^2^(*F*_o_^2^) + (0.0428*P*)^2^ + 0.7055*P*] where *P* = (*F*_o_^2^ + 2*F*_c_^2^)/3
*R*[*F*^2^ > 2σ(*F*^2^)] = 0.036	(Δ/σ)_max_ < 0.001
*wR*(*F*^2^) = 0.101	Δρ_max_ = 0.45 e Å^−^^3^
*S* = 1.10	Δρ_min_ = −0.43 e Å^−^^3^
968 reflections	Extinction correction: SHELXL97 (Sheldrick, 2008), Fc^*^=kFc[1+0.001xFc^2^λ^3^/sin(2θ)]^-1/4^
65 parameters	Extinction coefficient: 0.0045 (3)

### Special details

**Table:**

Geometry. All e.s.d.'s (except the e.s.d. in the dihedral angle between two l.s. planes) are estimated using the full covariance matrix. The cell e.s.d.'s are taken into account individually in the estimation of e.s.d.'s in distances, angles and torsion angles; correlations between e.s.d.'s in cell parameters are only used when they are defined by crystal symmetry. An approximate (isotropic) treatment of cell e.s.d.'s is used for estimating e.s.d.'s involving l.s. planes.

### Fractional atomic coordinates and isotropic or equivalent isotropic displacement parameters (Å^2^)

**Table:**

	*x*	*y*	*z*	*U*_iso_*/*U*_eq_	
Br	0.12607 (5)	0.16873 (4)	0.02778 (4)	0.0766 (3)	
O2	0.0390 (2)	0.4700 (2)	0.13501 (17)	0.0603 (6)	
O1	0.2328 (3)	0.3492 (2)	0.21071 (17)	0.0605 (6)	
C4	0.2152 (4)	0.3552 (3)	0.0252 (2)	0.0588 (8)	
H4A	0.3256	0.3483	0.0083	0.071*	
H4B	0.1644	0.4092	−0.028	0.071*	
C3	0.0090 (4)	0.5542 (4)	0.2241 (3)	0.0672 (9)	
H3A	0.0354	0.5012	0.2848	0.081*	
H3B	−0.1015	0.5779	0.2273	0.081*	
C1	0.1982 (3)	0.4327 (3)	0.1255 (2)	0.0545 (7)	
C2	0.3958 (4)	0.3150 (4)	0.2203 (3)	0.0680 (10)	
H2A	0.4284	0.2583	0.1628	0.082*	
H2B	0.4125	0.2609	0.2819	0.082*	

### Atomic displacement parameters (Å^2^)

**Table:**

	*U*^11^	*U*^22^	*U*^33^	*U*^12^	*U*^13^	*U*^23^
Br	0.0951 (5)	0.0623 (4)	0.0725 (4)	−0.00632 (17)	−0.00192 (18)	−0.00707 (17)
O2	0.0530 (11)	0.0674 (14)	0.0606 (12)	0.0026 (10)	0.0035 (10)	−0.0047 (11)
O1	0.0656 (13)	0.0620 (13)	0.0539 (12)	0.0008 (10)	−0.0008 (11)	0.0060 (10)
C4	0.0597 (18)	0.0570 (18)	0.060 (2)	0.0007 (14)	0.0019 (14)	−0.0019 (14)
C3	0.0648 (19)	0.073 (2)	0.064 (2)	0.0056 (16)	0.0107 (17)	−0.0070 (17)
C1	0.0510 (15)	0.0600 (19)	0.0525 (17)	0.0049 (13)	0.0015 (13)	−0.0006 (13)
C2	0.072 (2)	0.069 (2)	0.063 (2)	0.0096 (16)	−0.0108 (17)	0.0074 (17)

### Geometric parameters (Å, °)

**Table:**

Br—C4	1.933 (3)	C4—H4B	0.97
O2—C1	1.409 (4)	C3—C2^i^	1.489 (5)
O2—C3	1.442 (4)	C3—H3A	0.97
O1—C1	1.404 (4)	C3—H3B	0.97
O1—C2	1.434 (4)	C1—C1^i^	1.558 (6)
C4—C1	1.518 (4)	C2—H2A	0.97
C4—H4A	0.97	C2—H2B	0.97
			
C1—O2—C3	112.5 (2)	H3A—C3—H3B	108.2
C1—O1—C2	113.7 (3)	O1—C1—O2	105.9 (2)
C1—C4—Br	113.2 (2)	O1—C1—C4	113.3 (3)
C1—C4—H4A	108.9	O2—C1—C4	107.0 (3)
Br—C4—H4A	108.9	O1—C1—C1^i^	110.3 (2)
C1—C4—H4B	108.9	O2—C1—C1^i^	109.8 (3)
Br—C4—H4B	108.9	C4—C1—C1^i^	110.3 (2)
H4A—C4—H4B	107.7	O1—C2—C3^i^	110.0 (3)
O2—C3—C2^i^	110.0 (3)	O1—C2—H2A	109.7
O2—C3—H3A	109.7	C3^i^—C2—H2A	109.7
C2^i^—C3—H3A	109.7	O1—C2—H2B	109.7
O2—C3—H3B	109.7	C3^i^—C2—H2B	109.7
C2^i^—C3—H3B	109.7	H2A—C2—H2B	108.2

Symmetry codes: (i) −*x*+1/2, −*y*+1, *z*.
